# Epidemiology of Tuberculosis in Immigrants in a Large City with Large-Scale Immigration (1991-2013)

**DOI:** 10.1371/journal.pone.0164736

**Published:** 2016-10-17

**Authors:** Jesús E. Ospina, Àngels Orcau, Joan-Pau Millet, Miriam Ros, Sonia Gil, Joan A. Caylà, Barcelona Tuberculosis Immigration Working Group

**Affiliations:** 1 Servicio de Epidemiología, Agencia de Salud Pública de Barcelona, Barcelona, Spain; 2 Departamento de Pediatria, Ginecología y Medicina Preventiva, Universitat Autònoma de Barcelona (UAB), Barcelona, Spain; 3 CIBER de Epidemiología y Salud Pública (CIBERESP), Barcelona, Spain; National Institute of Health, ITALY

## Abstract

**Background:**

The increase in immigration in Barcelona between 2000 and 2008 forced a reorganization of the control of tuberculosis (TB). TB clinical units (TBCU) were created and community health workers (CHW) were gradually included.

**Objective:**

To understand trends in the incidence of TB among immigrants, their main characteristics and treatment compliance during the period 1991–2013.

**Design:**

We conducted a cross-sectional population-based study of cases detected among immigrants by the Tuberculosis Program in Barcelona, Spain. Sociodemographic, clinical characteristics and risk factors were described. The annual incidence was calculated for various periods and geographical areas of origin. In the linear trend analysis, a p-value of <0.05 was considered statistically significant.

**Results:**

We detected 3,284 cases. Incidence decreased from 144.8/100,000 inhabitants in 1991 to 53.4/100,000 in 2013. Individuals born in Pakistan-India-Bangladesh had the highest average annual incidence (675/100,000). In all, 2,156 cases (65.7%) were male. 2,272 (69.2%) had pulmonary TB, of which 48.2% were smear-positive. 33% of the cases (1,093) lived in the inner city. Contact tracing (CT) coverage in smear-positive individuals rose from 56.8% in 1991–1999 to 81.4% in 2000–2013 (p<0.01); this value was less than 50% in people from Africa and Eastern European countries. The case fatality rate was 3.6% overall and 9.8% among those born in high-income countries (p<0.01). The highest rate of treatment default (12.8%) was observed among cases from the Maghreb. The rate of successful treatment increased from 69.9% in 1991–1999 to 87.5% in 2000–2013 (p<0.01).

**Conclusion:**

The incidence of TB in immigrants is decreasing in Barcelona. Organizational actions, such as incorporating CHWs and TBCUs, have been decisive for the observed improvements.

## Introduction

In many high-income countries (HIC), migration from countries with a high burden of tuberculosis (TB) has gradually increased, modifying the local epidemiology and making control difficult [1]. In some European cities disease rates have increased dramatically due to social problems and limitations in disease control activities [1]. In cities such as London, an increase of more than 50% was recorded between 1999 and 2009 [2, 3]. Despite the decreasing trend in incidence, the decrease in the number of TB cases among the native population in some countries has often been accompanied by an increase in foreign-born populations [4, 5]. In Spain, overall incidence, including that in immigrants, remains higher than in many industrialized countries [6, 7].

In January 2015, 4,718,864 foreigners were registered in Spain (10.1% of the total population), whereas this percentage was only 1.8% in 1999, representing an increase of almost four million people in 16 years [8]. These percentages are even higher in cities such as Barcelona and Madrid, where the immigrant population has come to represent 16.3% and 12.1% of the total population, respectively [9,10]. Although the rate of immigration has declined because of the economic crisis, this decline has been very modest in Barcelona, only 0.4% lower in 2014 than in 2013 (5,345 fewer people) [9].

This demographic change has had a major impact on the epidemiology of TB in Barcelona, resulting in a slower reduction in incidence [11]. However, it has been possible to maintain a high percentage of successful treatment (ST), with no major differences between natives and immigrants. However, contact tracing (CT) studies have been performed in 88% of native smear-positive patients, but in less than 50% of the immigrant population [12]. Since 2003, the Barcelona TB Control Programme (PPCTB), with the incorporation of community health workers (CHW), has taken strategic measures to support the public health nursing team (PHNT). This has improved action protocols for immigrant populations in terms of monitoring cases and their contacts in accordance with international guidelines [13].

The aim of this study was to describe trends in the TB incidence, its main characteristics and treatment compliance (TC), in a city where the impact of immigration from countries with high incidence has been very significant and concentrated over a short time span of a few years.

## Methods

### Design and study population

A population-based cross-sectional descriptive observational study was performed. Both globally and for specific groups, cases born outside Spain who began treatment between January 1991 and December 2013, who were registered by the PPCTB, and who were resident in Barcelona were studied.

### Case definition

For the purposes of epidemiological surveillance and according to the European surveillance network’s criteria, TB case were defined as any patient who is prescribed TB treatment and complied with this until the expected time of completion, except if they died or presented serious side effects. Patients who restart TB treatment after more than one year are reincorporated into the case registry. Patients being treated for non-tuberculous mycobacteria are not considered cases [14].

### Definition of immigrant

Person born in a foreign country.

### Definition of geographical area of origin (Area of origin)

Cases were grouped into the following seven geographical areas based on their country of origin and their representation in the city: Latin America, India-Pakistan-Bangladesh, the Maghreb, high-income countries, Eastern Europe, the rest of Africa, and the rest of Asia.

### Variables

We included the following socio-demographic variables (sex, age, area of origin, and district of residence), risk factors (homelessness, imprisonment, alcoholism, injecting drug use (IDU), HIV infection, and smoking), and clinical characteristics (type of TB, radiology, multidrug-resistance (MDR), resistance to rifampin and isoniazid, CT, directly observed treatment (DOT), and treatment outcome (TO): cured, treatment completed, died, lost to follow-up, transferred, other. The rate of successful treatment (ST) is defined as the sum of cured individuals plus those who completed treatment divided by the total number of patients, expressed as a percentage.

### Statistical Analysis

Rates were calculated per 100,000 population. To calculate incidence rates, the denominator was the population of Barcelona born outside Spain, and also according to their geographic area of origin, as recorded in the city’s municipal census during the entire time series (1991–2013) [15].

We performed a descriptive analysis for the different variables by calculating proportions. Age as a quantitative variable was characterized by the median and interquartile range. Categorical variables were compared using the χ^2^ test. A p-value of <0.05 was considered statistically significant. The trend in the time series was calculated by fitting a line, and a p-value of <0.05 was considering statistically significant. All analyses were performed using the SPSS Statistical Package, version 13.0 (SPSS Inc, Chicago, IL, USA).

## Results

### Number of cases, distribution according to age, sex and sociodemographic characteristics

**Fig 1:**
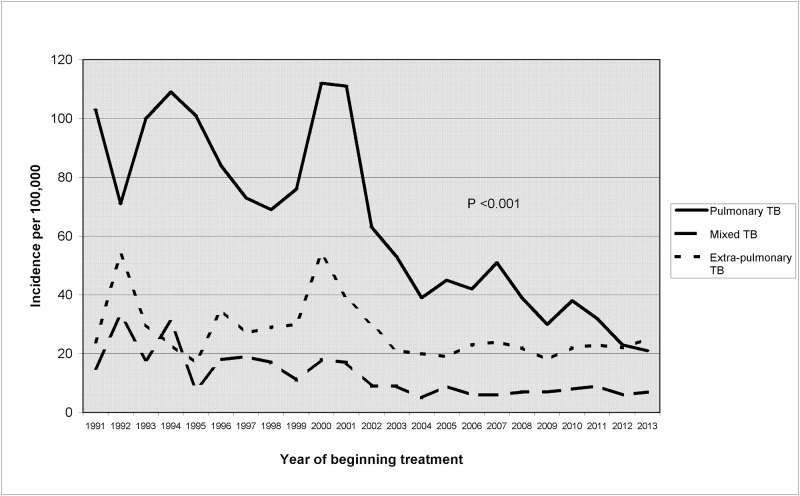
Trends in the incidence of pulmonary, mixed and extra-pulmonary tuberculosis in immigrants, Barcelona 1991–2013.

3,284 cases of TB were detected in immigrants residing in the city. Most came from Latin America (37.3%) and Pakistan, India and Bangladesh (26.6%). The average annual incidence was 105.9/100,000, decreasing from 144.8/100,000 in 1991 to 53.4/100,000 in 2013, representing an overall decline of 40.8%, and an average annual decline of 3.5% since 2000. This decline in incidence was observed in all clinical forms (Fig 1).

Table 1 shows the incidence of TB in the period from 1991 to 2013 according to geographical area of origin, and highlights the preponderance of men (65.7%; p <0.001), except among those from Latin America, where the percentage in each sex was similar. According to age group, the highest percentage was observed in individuals aged 25–44 years, with a similar distribution in men and women (40.7% and 41.1%, respectively). The Ciutat Vella district had a higher burden of disease (33.3%), especially among people from Pakistan, India and Bangladesh (Table 1).

**Table 1:** Main demographic characteristics of tuberculosis in immigrants, Barcelona, 1991–2013.

Year:	**1991**	**1992**	**1993**	**1994**	**1995**	**1996**	**1997**	**1998**	**1999**	**2000**	**2001**	**2002**
	**N(%)**	**N(%)**	**N(%)**	**N(%)**	**N(%)**	**N(%)**	**N(%)**	**N(%)**	**N(%)**	**N(%)**	**N(%)**	**N(%)**
New cases of TB	**66**	**76**	**75**	**85**	**66**	**75**	**64**	**71**	**80**	**137**	**170**	**153**
**Sex:**												
Male	48(72,7)	65(85,5)	56(74,7)	60(70,6)	51(77,3)	42(56,0)	37(57,8)	47(66,2)	50(62,5)	87(63,5)	127(74,7)	106(69,3)
**Age years:**												
0–14	0(0,0)	0(0,0)	1(1,3)	0(0,0)	0(0,0)	0(0,0)	0(0,0)	1(1,3)	0(0,0)	3(2,2)	1(1,3)	3(2,2)
15–24	7(10,7)	17(22,6)	17(22,6)	9(10,6)	7(10,6)	11(14,7)	15(23,5)	16(22,5)	12(15,1)	34(25)	37(21,8)	35(22,9)
25–44	45(69,3)	51(68)	41(56,6)	60(70,6)	47(71,2)	52(69,3)	40(62,5)	41(57,7)	58(72,5)	81(59,6)	105(61,7)	97(63,4)
45–64	8(12,3)	5(6,8)	12(16,0)	13(15,3)	12(18,2)	6(8,0)	8(12,5)	7(9,9)	9(11,3)	14(10,3)	17(10,0)	16(10,5)
≥65	5(7,7)	2(2,6)	4(5,3)	3(3,5)	0(0,0)	6(8,0)	1(1,6)	6(8,4)	1(1,3)	4(2,9)	10(5,9)	2(1,4)
**GAO:**												
Latin America	145	124	140	168	102	155	113	104	99	129	126	64
Pak-Ind-Ban	1565	3125	608	875	404	729	687	638	765	1621	948	546
Maghreb	158	128	394	258	220	114	120	161	170	165	296	170
HIC	38	68	47	52	87	66	33	37	20	57	22	35
Eastern E	190	174	0	461	296	277	0	318	175	339	124	139
Rest of Africa	770	491	394	309	264	346	980	390	491	493	625	136
Rest of Asia	164	152	197	337	145	158	118	162	193	247	132	91
**Municipal D:**												
Ciutat Vella	26(39,4)	35(46,1)	34(45,3)	31(36,5)	23(34,8)	24(32,0)	21(32,8)	24(33,8)	25(31,3)	62(45,3)	60(35,3)	62(45,3)
**Year:**	**2003**	**2004**	**2005**	**2006**	**2007**	**2008**	**2009**	**2010**	**2011**	**2012**	**2013**	**Total**
	**N(%)**	**N(%)**	**N(%)**	**N(%)**	**N(%)**	**N(%)**	**N(%)**	**N(%)**	**N(%)**	**N(%)**	**N(%)**	**N(%)**
**New cases of TB**	164	146	188	204	230	216	191	233	221	183	190	3284
**Sex:**												
Male	100(61,0)	98(67,1)	133(70,7)	128(62,7)	145(63,0)	132(61,1)	111(58,1)	158(67,8)	142(64,3)	120(65,6)	113(59,5)	2156(65,7)
**Age years:**												
0–14	4(2,4)	4(2,7)	7(3,7)	2(1,0)	1(0,4)	3(1,4)	3(1,6)	0(0,0)	2(0,9)	0(0,0)	3(1,6)	38(1,2)
15–24	32(19,5)	26(17,8)	32(17,0)	35(17,2)	44(19,2)	56(26,0)	32(16,8)	46(19,8)	51(23,1)	27(14,7)	28(14,7)	626(19,1)
25–44	105(64,0)	99(67,8)	126(67,0)	128(62,8)	151(65,7)	126(58,3)	130(68,1)	147(63,1)	128(57,9)	119(65,0)	123(64,7)	2100(64,0)
45–64	16(12,8)	11(7,6)	20(10,7)	32(15,7)	28(12,2)	30(13,8)	24(12,5)	35(15,1)	34(15,4)	34(18,6)	28(14,8)	424(12,9)
≥65	2(1,2)	6(4,2)	3(1,6)	7(3,5)	6(2,6)	1(0,5)	2(1,0)	5(2,1)	6(2,7)	3(1,6)	8(4,3)	93(2,9)
**GAO:**												
Latin America	61	48	56	56	72	57	48	50	41	32	28	
Pak-Ind-Ban	315	268	298	294	288	289	170	354	263	223	252	
Maghreb	152	75	101	93	109	44	73	104	114	76	83	
HIC	34	13	12	17	15	10	9	3	5	3	6	
Eastern E	48	123	82	71	78	72	96	62	97	44	67	
Rest of Africa	147	171	242	112	284	145	185	181	155	123	136	
Rest of Asia	77	41	61	78	58	77	37	53	28	48	53	
**Municipal D:**												
Ciutat Vella	65(39,6)	48(32,9)	55(29,3)	62(30,4)	71(30.9)	61(28,2)	41(21,5)	84(36,1)	59(26,7)	55(30,1)	65(34,2)	1093(33,3)

TB, tuberculosis; GAO, Geographic area of origin. For this variable, we show the annual incidence per 100.000 inhabitants; Pak-Ind-Ban, Pakistan-India-Bangladesh; HIC, High-income countries; Eastern E, Eastern Europe; Municipal D, Municipal district.

**Fig 2:**
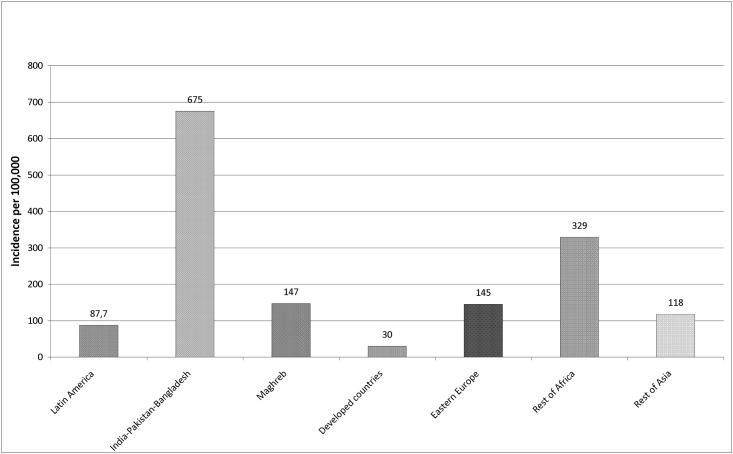
Average annual incidence of tuberculosis in immigrants according to geographic area of origin. Barcelona 1991–2013.

The highest average incidence was recorded among individuals from Pakistan, India, Bangladesh (675/100,000), followed by those from Africa (329/100,000), while that among individuals from high-income countries was only 30/100,000 (Fig 2).

**Fig 3:**
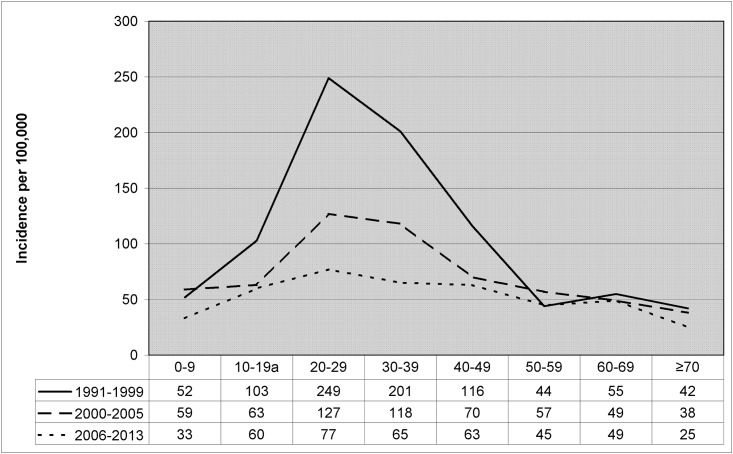
Average annual incidence of tuberculosis in immigrants according to age group and period, Barcelona 1991–2013.

In relation to age, over 70% of cases were aged 20–49 years and the maximum incidence was observed among 20- to 29-year-olds. Notably, the average annual incidence in this age group decreased from 249/100,000 in the early years of the study to 77/100,000 in recent years (Fig 3).

### Risk factors and clinical features

According to area of origin, cases from Eastern European countries and high-income countries had a higher frequency of risk factors such as a higher proportion of homelessness, history of imprisonment, alcoholism and smoking (Table 2).

**Table 2:** Risk factors for immigrants with tuberculosis, Barcelona, 1991–2013.

Geographic area	Latin America	Ind-Pak-Bang	Maghreb	HIC	Eastern E	Rest of Africa	Rest of Asia	Total
	N(%)	N(%)	N(%)	N(%)	N(%)	N(%)	N(%)	N(%)
**Cases of TB**	1224(37,3)	874(26,6)	360(11,0)	173(5,3)	198(6,0)	199(6,1)	256(7,8)	3284(100)
**Homelessness**	50(4,1)	47(5,4)	63(17,5)	23(13,3)	63(31,8)	27(13,6)	16(6,3)	289(8,8)
**Prison**	24(2,0)	5(0,6)	43(11,9)	18(10,4)	28(14,1)	22(11,1)	14(5,5)	154(4,7)
**Alcoholism**	152(12,4)	61(7,0)	71(19,7)	48(27,7)	58(29,3)	37(18,6)	32(12,5)	459(14,0)
**IDU**	32(2,6)	5(0,6)	19(5,3)	46(26,6)	39(19,7)	15(7,5)	12(4,7)	168(5,1)
**HIV**	145(11,8)	5(0,6)	25(6,9)	60(34,7)	31(15,7)	50(25,1)	12(4,7)	328(10,0)
**Smoking**	271(22,1)	199(22,8)	162(45,0)	96(55,5)	126(63,6)	71(35,7)	67(26,2)	992(30,2)
**Diabetes**	26(2,1)	23(2,6)	16(4,4)	3(1,7)	4(2,0)	2(1,0)	17(6,6)	91(2,8)

Ind-Pak-Bang, India-Pakistan-Bangladesh; HIC, High-income countries; Eastern E, Eastern Europe; IDU, Injecting drug use.

Regarding clinical characteristics, 58.1% of cases (n = 1909) had pulmonary TB, with the highest frequency observed among Eastern Europeans (77.3%). The most common type of extrapulmonary TB was lymphatic, with 638 cases (19.4%), followed by miliary TB, with 169 cases (5.1%), and osteoarticular TB, with 81 cases (2.5%). Notably, patients from the Indian subcontinent had a high percentage of extrapulmonary forms (49.8%), with lymphatic TB being the most common form in this group (337 cases, 38.6%). Latin Americans and Eastern Europeans had more cavitated and smear-positive forms People from Eastern Europe also showed more resistance to rifampicin (5.8%) and isoniazid (17.5%), and more MDR (4.5%) (Table 3).

**Table 3:** Clinical characteristics and indicators of tuberculosis control in immigrants, Barcelona, 1991–2013.

Geographic area	Latin America	Ind-Pak-Bang	Maghreb	HIC	Eastern E	Rest of Africa	Rest of Asia	Total
**Cases of TB**	1224(37,3)	874(26,6)	360(11,0)	173(5,3)	198(6,0)	199(6,1)	256(7,8)	3284(100)
**TB localization:**								
Pulmonary	798(65,2)	319(36,5)	240(66,7)	125(72,3)	153(77,3)	113(56,8)	161(62,9)	1909(58,1)
Extrapulmonary	294(24)	435(49,8)	87(24,2)	29(16,8)	29(14,6)	61(30,7)	68(26,6)	1003(30,5)
Both	129(10,5)	119(13,6)	31(8,6)	18(10,4)	15(7,6)	25(12,6)	26(10,2)	363(11,1)
**Radiology:**								
Normal	30(3,2)	25(5,7)	11(4,1)	7(4,9)	4(2,4)	8(5,8)	6(3,2)	91(4,0)
A. cavited	319(34,4)	114(26,0)	107(39,5)	44(30,8)	58(34,5)	47(34,1)	45(24,1)	734(32,3)
A. non- cavited	573(61,8)	297(67,8)	148(54,6)	91(63,6)	106(63,1)	82(59,4)	134(71,7)	1431(63,0)
**Smear-positive:**								
Pulm TB smear BK +	501(54,0)	149(34,0)	128(47,2)	82(57,3)	98(58,3)	70(50,7)	66(35,3)	1094(48,2)
**Drug resistance:**								
Isoniazid	79(8,5)	46(8,7)	14(6,3)	6(5,0)	27(17,5)	14(10,4)	20(12,0)	206(9,2)
Rifampicin	35(3,8)	15(2,8)	4(1,8)	3(2,5)	9(5,8)	5(3,7)	4(2,4)	75(3,3)
**MDR**	31(2,5)	13(1,5)	4(1,1)	2(1,2)	9(4,5)	5(2,5)	4(1,6)	68(2,1)
**DOT**	203(16,6)	240(27,5)	111(30,8)	35(20,2)	87(43,9)	69(34,7)	49(19,1)	794(24,2)
**CT** (S.P. BK+):								
Done	422(84,2)	114(76,5)	67(52,3)	55(67,1)	71(72,4)	47(67,1)	53(80,3)	829(75,8)
**Letality**	45(3,7)	15(1,7)	13(3,6)	17(9,8)	14(7,1)	7(3,5)	7(2,7)	118(3,6)
**ST**	1074(87,7)	747(85,5)	275(76,4)	134(77,5)	149(75,3)	159(79,9)	220(85,9)	2758(84,0)

Ind-Pak-Bang, India-Pakistán-Bangladesh; HIC, high-income countries; A. cavitated, abnormal cavitated; A. non-cavitated, abnormal non-cavitated; Pulm TB smear BK+, Pulmonar tuberculosis smear BK +; MDR, multidrug resistence; DOT, directly observed treatment; CT (S.P. BK+), contact tracing in smear-positive pulmonary patients; S.P., pulmonary smear-positive; bk+, smear-positive; ST, successful treatment.

### Outcomes of treatment and directly observed treatment

The rate of ST for the entire study period was 84%, with a 17.6% improvement between 1991–1999 (69.9%) and 2000–2013 (87.5%) (p<0.01). There was 14.3% loss to follow-up during the first period, decreasing to 4.7% during the second period (p<0.01). The cure rate increased from 1.2% to 28% among individuals from all geographic areas of origin from 2000 to 2013. Table 3 shows the percentage of patients in DOT for each geographical area.

Overall lethality was 3.6% and showed a decreasing linear trend during the study period (p<0.01). Lethality was 6,4% for the period 1991–1999, decreasing to 2.9% during the period 2000–2013. Results for each geographical area are shown in Table 3.

### Contact tracing (CT)

CT coverage increased from 56.8% in 1991–1999 to 81.4% in 2000–2013 (p<0.01). This percentage improved in all geographic areas during the second period, ranging from a 6.1% improvement in patients from Eastern Europe to a 30.6% improvement in those from Pakistan-India-Bangladesh. The lowest coverage was observed among Maghrebi patients and the highest among Latin Americans (Table 3).

## Discussion

In this study, we found that the incidence of TB among immigrants in Barcelona decreased significantly during the study period, with an average annual decline of 3.5% since 2000. Following the incorporation of CHWs and TBCUs, there has been a significant increase in ST and CT coverage.

While incidence remains high, it should be noted that much of the immigrant population arrived less than ten years ago. The Ciutat Vella district in the city centre, which is the most socio-economically disadvantaged and has a high prevalence of immigrants [2, 16], has the highest incidence (mean 125.9/100,000 in the last 11 years) and is the area where most of the population from Pakistan, India and Bangladesh have settled. Therefore, it is recommendable to maintain and improve active case-finding mechanisms, DOT and CT in all areas of social interaction [17].

As observed in New York [18], London [19] and Paris [20], TB is more common among men. Men may have a higher prevalence of latent TB infection and greater exposure to conditions that favor development of the disease, such as alcoholism, smoking and precarious working conditions [21, 22]. Among Latin Americans however the prevalence was similar among men and women (48.9% and 51.1%, respectively), possibly because Latin American migration to Spain has mainly been led by women [23, 24], unlike for other communities such as Pakistan and Maghreb. In terms of age group, the most affected were those between 15 and 44 years, similar to reports for immigrants in New York and London [18, 19]. In this young adult working-age population, the effects of the migration process may ultimately provoke development of the disease as a result of endogenous reactivation or recent infections in the destination country. It is important to note that incidence has declined in this age group over the years, and is now more similar that in natives. This profile of high TB incidence in individuals of working age corresponds to that of a country or large city that has still not achieved good control over the disease. The trend is positive however, considering that there has been a decrease over time in the incidence of recent infections in this age group (Fig 3) [25, 26].

While the observed incidence decreased during the study period thanks to better control and monitoring by the PPCTB, the number of cases among immigrants increased from the year 2000 onward. This is due to the massive influx of immigrants from low-income countries with a high incidence of TB. However, since 2008 the economic crisis may have contributed to lower immigration and thus a reduction in the number of cases. The highest incidence recorded during the entire period was observed in young adults from Pakistan, India and Bangladesh. This phenomenon may be associated with the high burden of disease and low socio-economic status of people from these countries. The overall rate among immigrants in 2013 (53.4/100,000) was higher than that observed among immigrants throughout the country for the same year (31.8/100,000) [27, 28], and much higher that reported for the entire EU population (12.7/100,000) [29]. In our case, the active surveillance carried out in the PPCTB would have an effect, allowing practically all cases diagnosed in the city to be registered.

The joint analysis of sex, age and risk factors such as homelessness, history of imprisonment, alcoholism, HIV infection, IDU and smoking, shows that men, young adults, and those from Eastern Europe, the Maghreb and high-income countries have a higher frequency of risk factors and thus greater vulnerability and management complexity [30, 31].

Pulmonary TB is the most common form in immigrants [18, 19, 20], as observed in this study. However, we observed a high percentage of cases with extrapulmonary forms from Pakistan, India and Bangladesh (49.8%), whose clinical management is more difficult and who contribute less to disease transmission. However, this percentage is higher (74.7%) for the whole of Spain, probably because in some regions not all cases of extrapulmonary TB are reported [32]. Regarding radiology, we observed a high frequency of cavitary forms in people from the Maghreb, Latin America, Eastern Europe, and the rest of Africa. This may be related to greater diagnostic delay, even among individuals from countries who speak the same language, such as those from Latin America.

The percentage of MDR-TB cases observed in immigrants in this study was much lower than for other regions [33, 34]. The percentage of resistance in people from Eastern Europe was especially high for isoniazid, reflecting the high prevalence of resistance in this region. There was a downward trend in the percentage of patients with TB/HIV, which did not appear to affect the epidemiology of TB in the city. This may be due to better control of coinfection, the increasing effectiveness of antiretroviral drugs, and the low prevalence of HIV infection in countries like Pakistan, India and Bangladesh which together with Latin America, represent 63.9% of all TB in this time series.

The percentage of treated and cured cases was high throughout the study period (84%). This percentage was almost equal to that observed in natives, and meets the current WHO targets for the EU. We also observed a significant improvement of 17% for the period 2000–2013, which was not the case for EU as a whole [13], or for neighboring regions. The percentage of cases lost to follow-up decreased by 9.6% during the second period.

A high percentage of the contacts of smear positive cases were investigated and treated, and we observed a 43.9% increase in coverage between 1991–1999 and 2000–2013. However, more work is needed to improve the exhaustivity of CT and ST in patients from Africa and high-income countries, who more often live alone or form part of groups at risk of social exclusion.

We observed a clear decline in lethality during this period, with an annual average of 1.9%. While the percentage was higher for high-income and Eastern European countries, lethality did not exceed 10% for any geographic area. Since 1987, the PPCTB and its PHNTs have achieved good indicators in monitoring and tracking patients and the contacts of native patients [12]. From 2000 onward, coinciding with the massive influx of immigrants from endemic countries [35], and following international [36, 37, 38, 39, 40] and national [41] recommendations, the program has gradually incorporated CHWs that act in coordination with PHNTs, doctors, technicians and other social and health agents, especially those in the TBCUs. The actions of the CHWs have helped improve treatment adherence, the search and location of patients and their contacts, access to the health card for free care, and control of outbreaks in family, work and leisure contexts [42, 43]. However, there have been considerable difficulty in monitoring and tracking contacts from Eastern Europe and the Maghreb.

This is one of the few studies that have been conducted in the immigrant population in a major European city. It is a population-based epidemiological study with a long follow-up period, and is based on an extensive, very reliable and refined TB database that has systematically collected data on the country of origin of each patient since the beginning of the PPCTB in 1987. However, this study has various limitations, including difficulty in precisely determining the denominators of the immigrant populations. The city’s municipal register does not exhaustively record immigrants country of origin, and many irregular immigrants, fearing deportation, probably do not fully disclose their data to the public administration. Hence, these incidence rates should be considered an approximation to reality because the available register may not accurately reflect the number of residents born outside Spain. Also, the number of migrants at risk during the period 1991–1999 was lower than that for the period 2000–2013, which may have influenced the high rates reported in the first period. Another limitation of the study is related to risk factors, namely the fact that the program’s epidemiological survey does not collect data on BMI (body mass index), and therefore we could not provide results on this.

## Conclusion

Immigration, especially massive immigration, is a challenge for TB programs, which must adapt to immigrants profiles according to their regions of origin. The adaptation of the PPCTB to these new challenges has allowed significant progress in the control and surveillance of the disease in immigrant groups over time. However, the results of our study demonstrate the need to continue encouraging monitoring and control activities, especially in immigrants from endemic countries. There is a need to maintain and improve active case-finding and tracking systems for both cases and contacts. In our case this was possible through coordinated intervention by PHNTs, case managers, CHWs, clinical units, and other health stakeholders, as well by promoting research and development in TB [35].

## Ethics approval statement

Demographic and clinical data were obtained from an epidemiological questionnaire used by the PPCTB. TB is a notifiable disease and therefore it is mandatory to record patients’ names for interviewing. The data were processed and analyzed anonymously. The analysis was carried out retrospectively and the data involved were collected according to the protocol of the National Tuberculosis Program, approved by the Spanish Ministry of Health. Therefore, no ethical approval or informed consent of patients was required. All data were treated in strict confidence in accordance with the ethical principles of the 1964 Declaration of Helsinki, revised by the World Health Organization in Edinburgh, 2000 and the Spanish Organic Law 15/1999 on Data Protection. Declaration of Helsinki from the World Medical Association: http://www.isciii.es/htdocs/terapia/documentos/Declaración of Helsinki.pdf. Spanish Organic Law 15/1999 on Data Protection: http://noticias.juridicas.com/base_datos/admin/lo15-1999.html.
