# Correction: Epidemiology of Tuberculosis in Immigrants in a Large City with Large-Scale Immigration (1991-2013)

**DOI:** 10.1371/journal.pone.0238963

**Published:** 2020-09-03

**Authors:** Jesús E. Ospina, Àngels Orcau, Joan-Pau Millet, Miriam Ros, Sonia Gil, Joan A. Caylà, Barcelona Tuberculosis Immigration Working Group

There are errors in the Funding statement. The correct Funding statement is as follows: This study was supported by Instituto de Salud Carlos III through funding awarded to author JPM (project “PI13/02757”) (co-funded by European Regional Development Fund). The funders had no role in study design, data collection and analysis, decision to publish, or preparation of the manuscript.
